# Novel pathogenic variant (c.2947C > T) of the carbamoyl phosphate synthetase 1 gene in neonatal-onset deficiency

**DOI:** 10.3389/fnins.2022.1025572

**Published:** 2022-10-21

**Authors:** Ruimiao Bai, ALing He, Jinzhen Guo, Zhankui Li, Xiping Yu, JunAn Zeng, Yang Mi, Lin Wang, Jingjing Zhang, Dong Yang

**Affiliations:** ^1^Department of Neonatology, Northwest Women’s and Children’s Hospital, Xi’an, Shaanxi, China; ^2^Department of Obstetrics, Northwest Women’s and Children’s Hospital, Xi’an, Shaanxi, China; ^3^Genetics Center, Northwest Women’s and Children’s Hospital, Xi’an, Shaanxi, China; ^4^Medical Imaging Center, Northwest Women’s and Children’s Hospital, Xi’an, Shaanxi, China

**Keywords:** carbamoyl phosphate synthetase 1 (CPS1), carbamoyl phosphate synthetase 1 deficiency (CPS1D), urea cycle disorder, molecular diagnosis, next-generation sequencing (NGS)

## Abstract

**Background:**

Carbamoyl phosphate synthetase 1 deficiency (CPS1D) is a rare autosomal recessive urea cycle disorder characterized by hyperammonaemia. The biochemical measurement of the intermediate metabolites is helpful for CPS1D diagnosis; it however cannot distinguish CPS1D from N-acetylglutamate synthetase deficiency. Therefore, next-generation sequencing (NGS) is often essential for the accurate diagnosis of CPS1D.

**Methods:**

NGS was performed to identify candidate gene variants of CPS1D in a Asian neonatal patient presented with poor feeding, reduced activity, tachypnea, lethargy, and convulsions. The potential pathogenicity of the identified variants was predicted by various types of bioinformatical analyses, including evolution conservation, domain and 3D structure simulations.

**Results:**

Compound heterozygosity of CPS1D were identified. One was in exon 24 with a novel heterozygous missense variant c.2947C > T (p.P983S), and another was previously reported in exon 20 with c.2548C > T (p.R850C). Both variants were predicted to be deleterious. Conservation analysis and structural modeling showed that the two substituted amino acids were highly evolutionarily conserved, resulting in potential decreases of the binding pocket stability and the partial loss of enzyme activity.

**Conclusion:**

In this study, two pathogenic missense variants were identified with NGS, expanding the variants pectrum of the *CPS1* gene. The variants and related structural knowledge of CPS enzyme demonstrate the applicability for the accurate diagnosis of CPS1D.

## Introduction

Carbamoyl phosphate synthetase 1 (*CPS1*) is a mitochondrial matrix enzyme involved in the first step of the urea cycle to convert bicarbonate (Shambaugh, 1977), ammonia, and adenosine triphosphate to carbamoyl phosphate (Summar, 1998; Yefimenko et al., 2005; Díez-Fernández et al., 2014; Ali et al., 2016). The *CPS1* gene (NM_001875.4) is located on chromosome 2q34 spanning approximately a 122 kb region. It consists of 38 exons, which encode 1,500 amino acids. To date, more than 240 natural mutations associated with CPS1D^1^ have been reported distributing into all exons of *CPS1* except exon 6. These variants include missense variants (59%), deletions (13%), gross deletions with splicing pathogenic variants (13%), and nonsense variants (7%) (Hoshide et al., 1993; Finckh et al., 1998; Summar, 1998; Ihara et al., 1999; Aoshima et al., 2001a; Rapp et al., 2001; Wakutani et al., 2004; Kurokawa et al., 2007; Häberle et al., 2011; Wang et al., 2011; Díez-Fernández et al., 2014; Ali et al., 2016; Choi et al., 2017). Nonsense variants would lead to truncated protein (i.e., c.2148 T > A, c.2033A > C), and variants at splicing sites leading to splicing variants (i.e., c.840G > C, c.860delA) (Martínez et al., 2010).

*CPS1* deficiency (CPS1D, OMIM#237300) is a rare autosomal recessive urea cycle disorder (UCD) (Ah Mew et al., 1993; Häberle et al., 2011). It is an unfortunately lethal inborn metabolism dysfunction. UCD indicates an interruption in the conversion of ammonia into urea, leading to deleterious hyperammonaemia (Nassogne et al., 2005; Gropman et al., 2007). CPS1D is classified into two types, neonatal-onset and delayed-onset. Most patients develop CPS1D within a few days after birth (neonatal-onset) and often deteriorate rapidly due to severe hyperammonaemia. The severity of the clinical manifestations of CPS1D is mainly dependent on the degree of enzymatic activity deficiency. Severe hyperammonaemia is often associated with a poor prognosis (Uchino et al., 1998; Funghini et al., 2012; Ali et al., 2016).

The diagnosis of CPS1D is primarily based on the biochemical measurement of intermediate metabolites, which cannot distinguish CPS1D from *N*-acetylglutamate synthetase deficiency (NAGSD) (OMIM 237310) (Häberle et al., 2003; Amstutz et al., 2011). Therefore, a method for urgent and accurate diagnosis of CPS1D is required. New techniques have become available for CPS1D diagnosis including next-generation sequencing (NGS) technology (Finckh et al., 1998; Aoshima et al., 2001b; Choi et al., 2017; Rokicki et al., 2017; Yang et al., 2017; Zhang et al., 2018). In this study, we performed a NGS to diagnose two gene variants of *CPS1* in a Chinese neonate with CPS1D. Remarkably, one of them is novel pathogenic variant in the *CPS1* gene for the first time.

## Materials and methods

### Patients, samples, and ethical approval

A 3-day-old boy was admitted to Northwest Women’s and Children’s Hospital on January 22, 2020 presenting with hypothermia and reduced activity. His main clinical manifestations and biochemical data are presented in Table 1 (Patient 8). An ethylenediaminetetraacetic acid (EDTA)-anticoagulated blood sample was obtained from the patient and both of his parents after informed consent for genetic tests. The confidentiality of the results was guaranteed. Approval for this study was granted by the Department of Academic Medical Affairs as well as by the institutional ethics committee of the Northwest Maternal’s and Children’s hospital.

**TABLE 1:** Summary of the clinical characteristics and mutational analyses of carbamoyl phosphate synthetase 1 deficiency (CPS1D) in Chinese patients.

Patient	Sex	Onset	Symptom	Ammonia (μg/dL)	Plasma citrulline (μmol/L)	Outcome	Hepatic Enzyme activity	Variants 1 (exon, amino acid change)	Variants 2 (exon, amino acid change)	Typeofvariants
P1 Yang et al., 2017	Female	Day 3	Tachypnoea, recurrent seizures	> 500	2.57	Death	NA	c.2407C > G (20.p.R803G)	c.323G > A (4.p.G108E)	Heterozygous variants
P2 Zhang et al., 2018	Male	Day 2	Poor feeding, vomiting	> 1,000	Low	Death on day 3	NA	c.2537C > T (p.P846L)	c.3443T > A (p.M1148L)	Heterozygous variants
P3 Zhang et al., 2018	Female	Day 5	Difficulty with feeding, reduced activity, dromsiness	739	Low	Death on day 8	NA	c.1799G > A (p.C600T)	12-bp deletion (c.4088_4099del, p. Leu 1363_Ile1366del)	Heterozygous variants
P4 Summar et al., 2013	Female	Day 4	Low activity, Tachypnoea	> 1,000	NA	Death	NA	c.173G > T (2.p.G58V)	c.796G > A (8.p.G266R)	Heterozygous variants
P5 Chen et al., 2013	Female	Day 2	Hyporeactiveness Respiratory distress; Seizures	1,404	3.82	Death on day 5	NA	c.1631C > T (p.T544M)	c.1981G > T (17.p.G661C)	Heterozygous variants
P6 Chen et al., 2013	Female	Day 3	Hyporeactive Grunting	823	3.08	Death on day 4	NA	c.2896G > T (p.E966X)	C622-3C > G	Heterozygous variants
P7 Chen et al., 2013	Female	Day 2	Low activity	217	NA	Death	NA	c.4357C > T (p.A1453T)		NA
P8	Male	Day 2	Hypothermia, Poor feeding, lethargy, seizures		4.34	Death	NA	c.2947C > T (p.P983S)	2548C > T (p.R850C)	Heterozygous variants

NA: data is not available.

### Genotyping by next-generation sequencing

The proband DNA was sequenced to discover the causal gene. DNA was isolated from the peripheral blood using the CWE9600 Automated Nucleic Acid Extraction System with the CWE2100 Blood DNA Kit V2 (CWBiotech, Beijing, China, CW2553). The genomic DNA (750 ng) was fragmented into 200–300 bp using the Scientz08-III Ultrasonic Homogenizer (SCIENTZ, Beijing, China). The DNA fragments were processed by end-repair, A-tailing, and adaptor ligation with the KAPA Library Preparation Kit (Illumina, KR0453, v3.13) and 8-cycle precapture polymerase chain reaction (PCR) amplification. The amplified DNA sample was captured in the Agilent SureSelect XT2 Target Enrichment System (Agilent, Technologies, Inc., Santa Clara, USA). The captured DNA fragments were purified using the DynabeadsMyOne Streptavidin T1 (Thermo Fisher Scientific, Waltham, USA) and amplified using 13-cycle postcapture PCR. The final products were purified using the AgencourtAMPure XP (Beckman Coulter, Brea, USA) and quantitated using the Life Invitrogen Qubit 3.0 by Qubit dsDNA HS Assay Kit (Thermo Fisher Scientific, Waltham, USA). Finally, the quantified DNA was sequenced using 150 bp paired-end reads on the Illumina Novaseq 6000 platform (Illumina, San Diego, USA) in accordance with the standard manual.

The raw data generated on the Novaseq platform were filtered and aligned against the human reference genome (hg19) using BWA Aligner^2^ after evaluation in accordance with the Illumina Sequence Control Software. Single-nucleotide polymorphisms (SNPs) were identified using the GATK software (Genome Analysis ToolKit^3^). The variants were annotated using ANNOVAR^4^. Moreover, the effects of single-nucleotide variants (SNVs) were predicted using SIFT^5^, Polymorphism Phenotyping v2 (Polyphen-2, I. (Adzhubei et al., 2010) and Variant Taster (Schwarz et al., 2010). All variants were interpreted in accordance with the American College of Medical Genetics and Genomics (ACMG) standards such as the categories of pathogenic, likely pathogenic, variants of unknown clinical significance, likely benign, and benign.

### Structural domain analysis

The protein sequence was obtained from Uniprot (ID:P31327)^6^. The Pfam domain database (^7^) was used for structural domain analysis and Lollipops was used to visualize the domain structure and highlight variants (Jay and Brouwer, 2016).

### Pathogenicity and conservation analysis

To predict the potential structural influence of detected amino acid substitution, we used physical and evolutionary comparative algorithms in Polyphen-2. Through Blast, the orthologous genes of *CPS1* were identified by simply comparing CPS1 protein query to a protein database. The evolution tree was drawn based on the gene orthology/paralogy prediction method implemented in the Ensemble database^8^. Multiple sequences were aligned using Muscle (Finckh et al., 1998; Summar, 1998; Edgar, 2004). The multiple-alignment sequence was used to create the sequence logo (seqlogo) using weblogo software^9^ (Crooks et al., 2004). We have also obtained VCF format for our variants. CADD score was obtain through the website^10^.

### Binding site prediction of long non-coding RNA

Human LncRNA sequences were downloaded from the Ensemble website (hg19), and formatdb was used to build the Blast search database (Altschul et al., 1990). The potential binding sites for *CPS1* were obtained using Blastn with an *e*-value cutoff of e-15. An in-house Python script was used to filter potential LncRNA interactions with complemental hits (plus/minus hits) and 80% sequence similarity as the threshold.

### Domain structure simulation and structural modeling analysis

*CPS1* and variant sequences were generated using the an-house Python script. The structure of 6UEL was obtained as a template from the Protein Data Bank (PDB) database^11^ and the Swiss-model server was used to model the 3D structure (Waterhouse et al., 2018). The simulated protein 3D structures of wild and variant *CPS1* were aligned using Superpose version 1.0 (Maiti et al., 2004). Protein super positions were calculated using the quaternion approach. Wild and variant structures were visualized using Rasmol version 2.7 (Sayle, 1994). The relative position of the variant site was determined based on the structural alignments.

**FIGURE 1:**
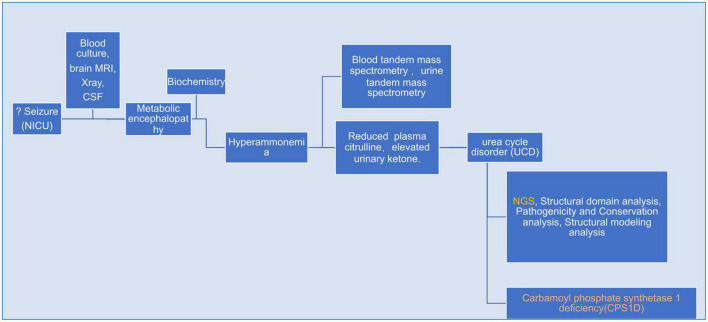
A flow-chart specifically describing bioinformatics & analysis process for the whole case.

A flow-chart summerizing bioinformatics & analysis process as showed in Figure 1.

## Results

### Clinical characteristics of the patient with Carbamoyl phosphate synthetase 1 deficiency

The patient was born at 40^+3^ weeks’ gestation age via spontaneous delivery with a birth weight of 3,400 g. No gross birth abnormality was identified. The prenatal care was unremarkable. The patient presented with poor feeding, reduced activity, tachypnoea, and lethargy with rapid progression on day 3 after birth. Additional symptoms included intermittent trembling of the extremities involving hypertonia and spasms of the upper limbs lasting for approximately 1 min. The patient did not respond well to the initial treatments, such as oxygen therapy, meropenem, and phenobarbital, and was then transferred to our neonatal intensive care unit (NICU). The patient’s body weight dropped to 3,110 g upon admission.

**FIGURE 2:**
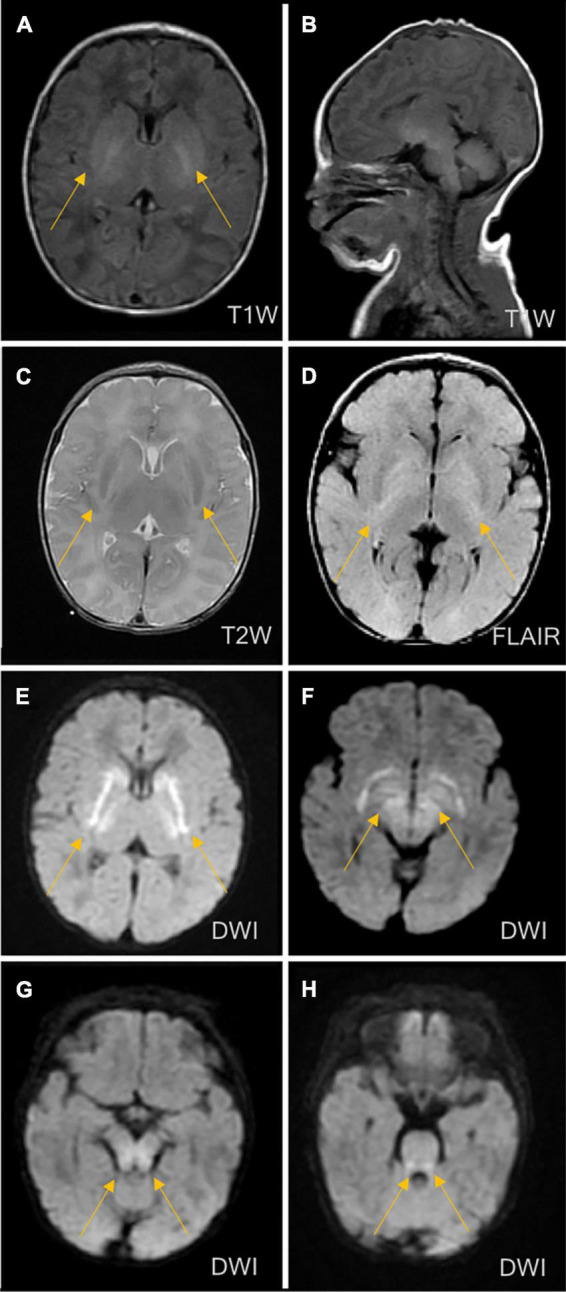
Brain magnetic resonance imaging (MRI) on the third day of life. MRI showed symmetrical diffuse high-signal shadows in the bilateral basal ganglia, cerebellum, pontine, tegmental and bilateral frontal and parietal cortices, suggesting hereditary metabolic leukoencephalopathy. **(A)** No abnormal signal intensity in the bilateral basal ganglia on the T1-weighted image (T1WI). **(B)** Low signal intensity in the bilateral basal ganglia on the T2-weighted image (T2WI). **(C)** High signal intensity in the bilateral basal ganglia on the diffusion-weighted image (DWI). **(D)** High signal intensity in the bilateral basal ganglia on fluid-attenuated inversion recovery (FLAIR). **(E)** High signal intensity in the cerebellum on the DWI. **(F)** High signal intensity in the pontine on the DWI. **(G)** High signal intensity in midbrain on DWI. **(H)** High signal intensity in medulla on DWI.

The patient’s vital signs (temperature, 36.8°C; heart rate, 138 bpm; and breath rate, 60 breaths per minute) were stable. Hypotonia of the extremities, coarse lung breath, and absence of a primitive reflex were observed during physical examination. Blood gas analysis showed respiratory alkalosis, but other laboratory measurements including peripheral blood counts, glucose, electrolytes, liver function tests, creatinine, and creatinine kinase were within the normal ranges. The coagulation function testing result indicated a mildly prolonged prothrombin time of 80 s. The blood culture result showed *Staphylococcus epidermidis*. The cerebrospinal fluid (CSF) examination was negative, and the chest X-ray revealed pneumonia. At this point, neonatal sepsis and clinic seizures were considered, and intracranial infection was also suspected. Therefore, vancomycin (based on the result of drug sensitivity to vancomycin), phenobarbital, and other supporting therapies, such as fasting, were administered. Further evaluation with brain magnetic resonance imaging (MRI) was ordered and showed symmetrical diffuse high-DWI signal intensity in the bilateral basal ganglia, cerebellum, pontine, bilateral frontal and parietal cortices with corresponding T2/FLARE signal hyperintensity, suggesting hereditary metabolic encephalopathy (Figure 2). Laboratory tests revealed serum ammonia of 350 μmol/L (normal range: 40–80 μmol/L). Blood tandem mass spectrometry revealed reduced serum citrulline (4.34 mmol/L, normal range: 5–30 mmol/L), and urine tandem mass spectrometry showed elevated urinary ketone. UCD was therefore considered. On the seventh day after birth, rapid deterioration with hypotonia and lethargy were observed and mechanical ventilation support was thus provided. After long discussion, the parents decided to terminate treatment due to poor prognosis. The infant passed away at home after discharged against medical advice on day eight after birth.

### Detection of two heterozygous variants

**FIGURE 3:**
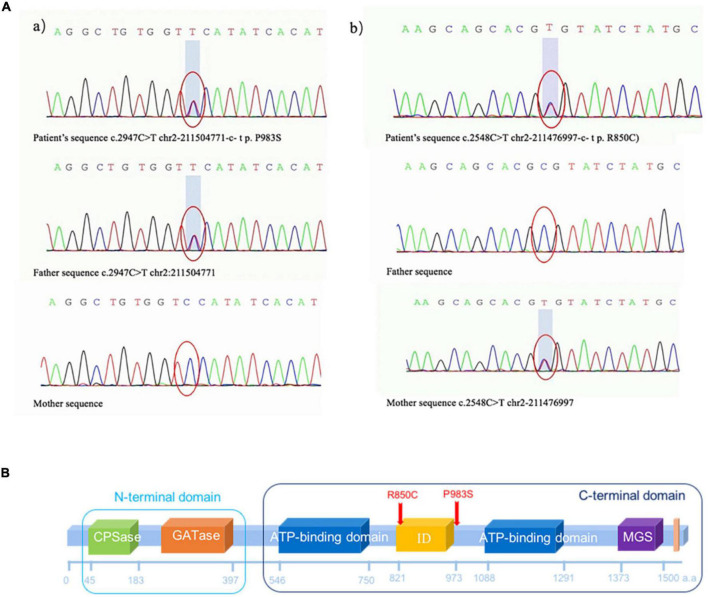
**(A)** Missense variant identified in the *CPS1* gene in the patient through exome sequencing. (a) Novel heterozygous missense variants c.2947C > T (p.P983S) in exon 24 and (b) c.2548C > T (p.R850C) in exon 20. (b) Sanger sequencing revealed that the father had a heterozygous missense variant c.2947C > T (p.P983S), and the mother had a heterozygous missense variant c.2548C > T (p.R850C). **(B)** The CPS1 p.R850C variant was located in the CPS large-chain oligomerization domain (http://pfam.xfam.org/protein/P31327). The *CPS1* p.P983S variant was not present in the protein structural domain. 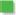 Carbamoyl phosphate synthetase (CPS) small chain, CPSase domain. The CPS domain is in the amino terminus of the protein. CPS catalyses the ATP-dependent synthesis of carbamoyl phosphate from glutamine or ammonia and bicarbonate and initiates the urea cycle and the biosynthesis of arginine and/or pyrimidines. 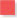 GATase: Glutamine amidotransferase class-I. Type 1 glutamine amidotransferase (GATase1)-like domain is found in formylglycinamide ribonucleotide amidotransferase (FGAR-AT). The GATase activity catalyses the transfer of ammonia from the amide side chain of glutamine to an acceptor substrate. 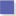 CPS Large chain, ATP-binding domain. CPS catalyses the ATP-dependent synthesis of carbamoyl phosphate from glutamine or ammonia and bicarbonate and initiates the urea cycle and the biosynthesis of arginine and/or pyrimidines (Simmer et al., 1990). The CPS enzyme in prokaryotes is a heterodimer of small and large chains. The small chain promotes the hydrolysis of glutamine to ammonia, which is used by the large chain to synthesize carbamoyl phosphate (Pfam:PF00988). The small chain has a GATase domain in the carboxyl terminus (Pfam:PF00117). The ATP-binding domain has an ATP–grasp fold. 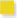 CPS ID, integration domain. CPS catalyses the ATP-dependent synthesis of carbamoyl phosphate from glutamine or ammonia and bicarbonate. The CPS enzyme in prokaryotes is a heterodimer of small and large chains. 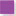 GS-like domain. The MGS-like domain comprises the entire protein of methylglyoxal synthetase, which catalyses the enolisation of dihydroxyacetone phosphate to produce methylglyoxal. The family also includes the C-terminal domain in CPS, which catalyses the last phosphorylation of a carboxyphosphate intermediate to form the product carbamoyl phosphate and may play a regulatory role. This family also includes inosine monophosphate cyclohydrolase. The known structures in this family have a common phosphate-binding site (Raushel et al., 1998, 2003; Holden et al., 1999). 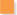 CPS, C-terminal domain. This is the C-terminal domain found after the MGS domain (Pfam:PF02142) in human CPS. CPS catalyses the first step of ammonia detoxification to urea (de Cima et al., 2015).

Two heterozygous missense variants c.2947C > T (p.P983S) in exon 24 (a) and c.2548C > T (p.R850C) in exon 20 (b) were identified in the patient through NGS (Figures 3A,B). Two variants were subsequently confirmed using Sanger sequencing. Additional family sequencing data showed that the father had a heterozygous missense variant c.2947C > T (p.P983S), while the mother had a heterozygous missense variant c.2548C > T (p.R850C). His brother’s genotype could not be confirmed because a sample could not be obtained. According to the ACMG guidelines (Richards et al., 2015), ClinVar^12^, and the Human Gene Variant Database (HGMD) (Stenson et al., 2017), two heterozygous SNPs of the *CPS1* gene were detected as potential pathogenic variants.

## Structural domain analysis, pathogenicity, and conservation analysis

**FIGURE 4:**
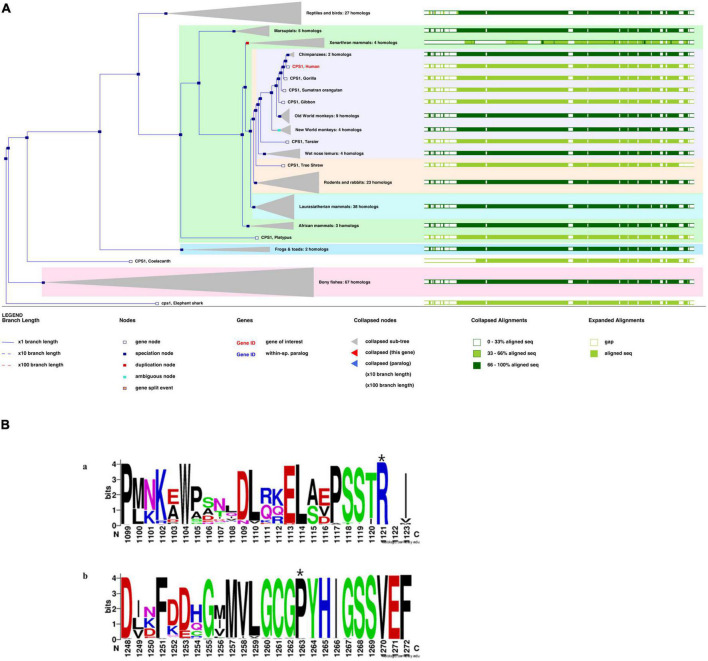
**(A)** Phylogenetic tree of CPS1 with 197 homologous sequences from 186 species. **(B)** The sequence logo includes stacks of symbols for the corresponding amino acids. The height of the stack reflected the conservation of amino acids. The chemical properties of the amino acids were used to define the color system. Polar (G, S, T, Y, C, Q, and N), basic (K, R, and H), acidic (D and E), and hydrophobic (A, V, L, I, P, W, F, and M) amino acids are shown in green, blue, red, and black, respectively(Li et al., 2021). Variant sites: **(A)** p.R850C at the 1,121st amino acid position above the seqlogo (marked with a star). **(B)** p.P983S at the 1,263rd amino acid position above the seqlogo (marked with a star).

As shown in Figure 4, the *CPS1* p.R850C variant was located in the CPS large-chain oligomerization domain^13^. The *CPS1* p.P983S variant was not present in the protein structural domain.

Based on functional effects analysis of the above variants using Polyphen-2, both variants were predicted as probably damaging with scores of 1.0 (sensitivity, 1.0; specificity, 1.0) and 0.94 (sensitivity, 0.8; specificity, 0.94), respectively (data now shown). CADD is also a great tool for scoring the deleteriousness of single nucleotide variants in the human genome and providing with a great value of predictions. The CADD was calculated as 7.89 (recommended threshold >23.95), which indicates that these are predicted to be less than 10% most deleterious substitutions that affect the human genome.

A phylogenetic tree for *CPS1* was generated with 197 homologous sequences from 186 species using the homolog from the elephant shark as an outgroup (Figure 4A). The greatest similarity was found between humans and primates. Most parts of the sequences were highly conserved (green bars, >33%).p.R850 is variant in vertebrate CPSs (judged from the 237 sequences alignment) and p.P983 is very rarely substituted by other residues (x substitutions among the 237 aligned sequences), with no occurrence of serine in these substitutions (Figure 4B). The variant site (p.P983S) was also the most conserved residue at this position. The S (Ser) variant was rare (Figure 4B; Li et al., 2021).

### Binding site prediction for LncRNAs and structural modeling

The Blast results showed possible binding of *CPS1* to 17 LncRNAs. However, the two variant sites were not located within the 275 potential binding regions. Therefore, the binding region was not overlapped with the variant position.

**FIGURE 5:**
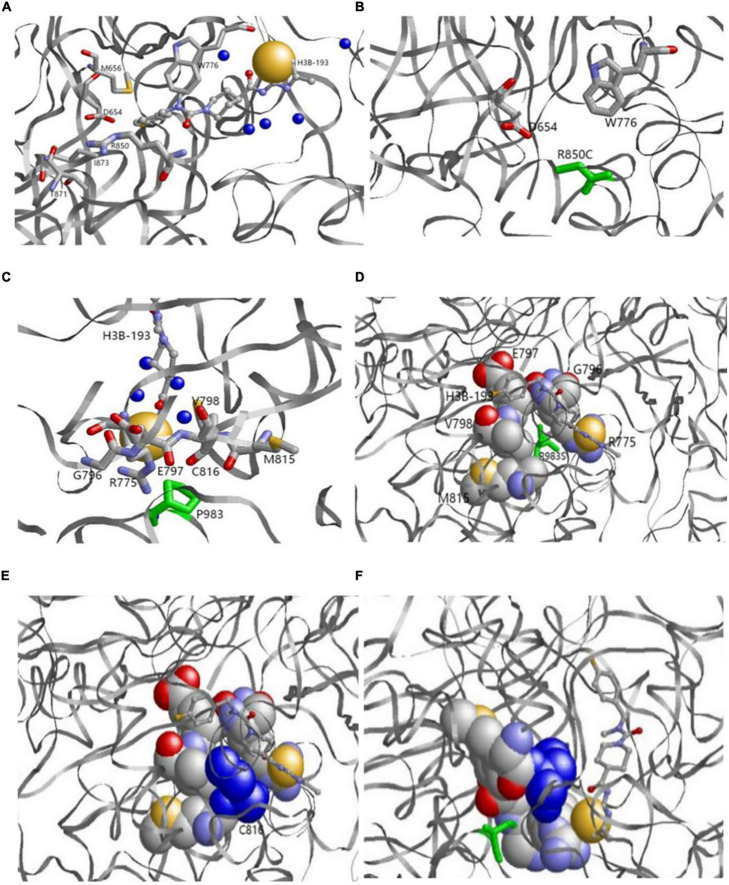
**(A,B)** Original (a) and variant (b) models of p.R850. **(A)** Original p.R850 located near the binding pocket binds the ligand (allosteric inhibitor H3B-193) as well as P.D654, p.M656, p.W776, p.T871, and p.I873. **(B)** Variantp.R850C (green) reduces the binding partner in the pocket. p.R850C only binds to p.E654 and p.W776**. (C,D)** Original p.P983model. p.P983 (green) is an important binding residue with part of the binding pocket containing residues p.R775, p.G796, p.E797, p.V798, p.M815, and p.C816. **(E,F)** Variantp.P983S model. Variantp.P983 (green) reduces the binding surface. p.C816 (blue) is a residue in the binding pocket and no longer binds with p.P983S. The left and right bottom images show the side views from the back.

The 3D structure showed that p.R850 was located near the binding pocket. In the 6UEL model, p.R850 was bound to the ligand (allosteric inhibitor H3B-193) as well as p.D654, p.M656, p.W776, p.T871, and p.I873 to form a stable binding pocket (Figure 5A). The variant p.R850C (a short residue C replaced a long residue R) reduced the binding partner in the pocket. Our model showed that p.R850C was only bound to p.E654 and p.W776 (Figure 5B).

p.P983 showed no variation, whereas p.G982, p.Y984, p.I986, and p.G987 had natural variants (See text footnote 1). The results demonstrated that p.P983 was an important binding residue with part of the binding pocket containing residues p.R775, p.G796, p.E797, p.V798, p.M815, and p.C816 (Figures 5C,D). The variant p.P983S also significantly reduced the binding surface in our model after variant. p.C816 was a residue in the binding pocket and no longer bound with p.P983S (Figures 5E,F).

## Discussion

The patient in the present study presented the clinical manifestations of urea cycle disorder (UCD). We have used NGS instead of single gene sequencing. In our case a specific variant or a target gene associated disease remain unclear, screening-based NGS would make more sense to provide sufficient information about candidates of single nucleotide variant or DNA variations within protein-coding regions. Two compound heterozygous variants of *CPS1*were found by NGS and *in silico* analysis, one of which is novel. The variants likely account for patient’s symptom. The patients with CPS1D typically exhibit elevated ammonia and decreased downstream production of urea (Häberle et al., 2012). The clinical manifestation is associated with neural function disturbances attributable to hyperammonaemia. CPS1D can be characterized as neonatal and late-onset according to the age of onset. Patients with neonatal-onset CPS1D are often normal at birth. The clinical manifestations include feeding difficulties, hypothermia, hypotonia, and seizure. The severity of the clinical manifestations of CPS1D depends on the degree of the enzymatic activity deficiency. The age of onset for neonatal-onset CPS1D ranges from day 0 to 20 (Hommes et al., 1969; Freeman et al., 1970; Ebels, 1972; Gelehrter and Snodgrass, 1974; Suzuki et al., 1986; Finckh et al., 1998; Kurokawa et al., 2007). Most patients with CPS1D present with neonatal onset and usually passed away quickly due to hyperammonaemia, leading to encephalopathy (Kido et al., 2012; Kölker et al., 2015). Patients with late-onset CPS1D manifest symptoms at different ages, ranging from 9 years to 45 years (Granot et al., 1986; Verbiest et al., 1992; Wong et al., 1994; Kurokawa et al., 2007). Hence, the treatment of CPS1D is very limited, which includes protein restriction, the use of nitrogen scavengers, and the reversal of endogenous (protein) catabolismand establishment of anabolism; gene therapy are under development (Diez-Fernandez and Häberle, 2017); liver transplantation is currentlythe only cure.

The exact incidence of CPS1D remains unknown but has been estimated as follows in several populations: 1/62,000 in the USA, 1/800,000 in Japan, and 1/539,000 in Finland (Brusilow and Maestri, 1996; Summar et al., 2008, 2013; Tuchman et al., 2008). To date, hundreds of CPS1D cases have been reported worldwide (Häberle et al., 2011). To our knowledge, the frequency of CPS1D has not been investigated in Chinese cohorts and only eight Chinese patients with CPS1D, including the patient in the present study, have been reported (Chen et al., 2013, 2018; Yang et al., 2017; Zhang et al., 2018; Yan et al., 2019). Table 1 summarizes the results of the present study together with those for seven previously reported Chinese patients. Chinese patients with CPS1D were reported to have early onset with average of 2.88 days of life (Table 1), consistent with previous reports (Hommes et al., 1969; Freeman et al., 1970; Ebels, 1972; Gelehrter and Snodgrass, 1974; Suzuki et al., 1986; Finckh et al., 1998; Kurokawa et al., 2007). Low plasma citrulline levels (average of 3.45 μmol/L) were found in all cases, associated with CPS1D. Patients with neonatal-onset CPS1D have extremely poor clinical outcome with 100% mortality and rapid decompensation (average of 5 days’ survival after birth) despite intensive therapy, including protein restriction and hemodialysis/peritoneal dialysis (Table 1). This disease is essentially lethal and detrimental, the exact etiology of neonatal-onset CPS1D in these Chinese patients is poorly understood. In our study, MRI (Figure 2) revealed symmetrical diffuse high T2/FLARE signal intensity with corresponding restricted diffusion in the bilateral basal ganglia, cerebellum, pontine, bilateral frontal and parietal cortices, likely associated with higher hyperammonaemia (Takanashi et al., 2003). Imaging findings for hyperammonaemia encephalopathy is otherwise non-specific. Due to the delayed diagnosis and limited life span, there is a lack of MRI brain data for CPS1D patients. There are no characteristic MRI finding to date for differentiating CPS1D from other causes for neonatal hyperammonaemia/urea cycle disorder (UCD).

*N*-acetylglutamate synthase deficiency (NAGSD) is another type of UCD, with clinical manifestation similar to those of CPS1D(Ali et al., 2016; Sancho-Vaello et al., 2016). Data for the hepatic enzyme activity in Chinese patients with CPS1D are unavailable since CPS1D is a rare disease with an atypical clinical presentation. Molecular genetic diagnosis of CPS1D is often required. In the present study, we successfully used NGS to identify a novel missense variant of c.2947C > T (p.P983S) in exon 24 and a previously known pathogenic variant of c.2548C > T (p.R850C) in exon 20 of the *CPS1* gene (Martínez et al., 2010; Díez-Fernández et al., 2014). The variant of c.2548C > T (p.R850C) has been reported in Japanese patients with CPS1D (Kurokawa et al., 2007). Furthermore, a family study with targeted pathogenic variant analysis of the two *CPS1*variants revealed that the patient’s father has a heterozygous missense variant c.2947C > T (p.P983S) whereas the patient’s mother has a heterozygous missense variant c.2548C > T (p.R850C). Structural domain analysis in the current study revealed that c.2548C > T (p.R850C) was located within the CPS large-chain oligomerization domain, whereas another novel variant c.2947C > T (p.P983S) was absent in the protein structural domain (Figure 3B). Moreover, the predicted pathogenicity showed that both variants were probably damaging with scores of 1.0 (sensitivity, 1.0; specificity, 1.0) and 0.94 (sensitivity, 0.8; specificity, 0.94). These results suggested that the sites were highly likely to be pathogenic variants related to CPS1D. Further conservation analysis indicated that the variant site c.2548C > T (p.R850C) was located at the 1,121st amino acid position, and Arg was 100% conserved in the evolution across various species. The C variant was absent in natural evolution (Figure 4A). Otherwise, the variant site c.2947C > T (p.P983S) was located at the 1,263rd amino acid position, and Pro was the most conserved residue at this position. The variant to Ser was rare (Figures 4A,B), suggesting that both amino acid substitutions occur at highly evolutionary conserved positions. There is also a predicted nuclear receptor binding site was around positions p.R850 and p.P983. LncRNAs are emerging as a class of important regulators that participate in various biological functions and disease processes. Our binding site prediction for LncRNAs suggested possible binding of the *CPS1* to 17 LncRNAs; however, the two variant sites were not located within the 275 potential binding regions, suggesting indirect interaction likely thorough these 17 LncRNAs or an enzyme complex.

Alternatively, the 3D structure showed that p.R850 was located near the binding pocket. In the 6UEL model, p.R850 binds the ligand (allosteric inhibitor H3B-193) as well as p.D654, p.M656, p.W776, p.T871, and p.I873 to form a stable binding pocket (Figure 5A). The variant c.2548C > T (p.R850 C, a short residue C replaces a long residue R) reduced the binding partner in the pocket. In our model, c.2548C > T (p.R850C) only bound to p.E654 and p.W776, indicating a low binding affinity to the molecule that fits in the pocket. This finding would potentially explain the partial loss of enzyme activity. We also found natural variants for p.G982, p.Y984, p.I986, and p.G987 but not for p.P983 (Figures 5C,D). p.P 983 is an important binding residue with part of the binding pocket containing residues p.R775, p.G796, p.E797, p.V798, p.M815, and p.C816, suggesting its role as a pocket stabilizer. The variant c.2947C > T (p.P983S) reduced the binding surface in our model.p.C816 was a residue in the binding pocket and no longer bound to c.2947C > T (p.P983S). Hence, p.C816 could affect the pocket stability (Figure 5D). Overall findings suggested the potential decreases of the binding pocket stability and the partial loss of enzyme activity due to those variants at two sites.

This study has a few limitations. The genotype of the patient’s brother should be further evaluated; however, the sample was unavailable because of the parents’ refusal. The segregation data through the extended family was not performed. The specificity of novel variant at c.2947C > T (p.P983S) for Chinese population was not yet tested. Further functional studies and downstream experiments are necessary to elucidate the molecular pathogenesis of the novel missense variant. Moreover, investigation of the genotype through genetic testing of next child and genetic counseling is not yet performed but suggested (Özcan et al., 2017).

In conclusion, we represented a comprehensive clinical case report of a neonate with clinical features consistent with carbamoyl phosphate synthetase 1 deficiency (*CPS1*D). NGS identified balletic rare variants of *CPS1* that likely explain the proband’s condition. Our findings expand the variantal spectrum of *CPS1*.

## Data availability statement

The raw data supporting the conclusions of this article will be made available by the authors, without undue reservation.

## Ethics statement

The studies involving human participants were reviewed, approved by the Department of Academic Medical Affairs as well as by the Institutional Ethics Committee of the Northwest Maternal’s and Children’s Hospital. All methods were carried out in accordance with relevant guidelines and regulations. Written informed consent to participate in this study was provided by the participants or their legal guardian/next of kin.

## Author contributions

RB, AH, DY, and ZL conceptualized and designed the study. RB, AH, DY, and JG acquired the data. RB, LW, and YM analyzed the data. RB, JG, JJZ, JAZ, and XY interpreted the results. RB drafted the manuscript. All authors reviewed and revised the manuscript for important intellectual content and approved the final manuscript as submitted and agreed to be accountable for all aspects of the work.

## Footnotes

1
https://www.uniprot.org/uniprot/P31327

2
http://bio-bwa.sourceforge.net/

3
www.broadinstitute.org/gatk

4
https://annovar.openbioinformatics.org/en/latest/

5
https://sift.bii.a-star.edu.sg/

6
https://www.uniprot.org/

7
http://pfam.xfam.org/

8
http://asia.ensembl.org

9
http://weblogo.berkeley.edu/

10
https://cadd.gs.washington.edu/info

11
https://www.rcsb.org/structure/6uEL

12
https://www.ncbi.nlm.nih.gov/clinvar/

13
http://pfam.xfam.org/protein/P31327

## Abbreviations

CPS1: Carbamoyl phosphate synthetase 1
CPS1D: Carbamoyl phosphate synthetase 1 deficiency
NGS: Next-generation sequencing
UCD: Urea cycle disorder
NASD: N-acetylglutamate synthetase deficiency
EDTA: Ethylenediaminetetraacetic acid
SNPs: Single-nucleotide polymorphisms
ACMG: American College of Medical Genetics and Genomics
LncRNA: long non-coding RNA
PDB: Protein Data Bank
NICU: Neonatal intensive care unit
CSF: Cerebrospinal fluid
MRI: Magnetic resonance imaging.
